# Effects of an Essential Oil Blend on In Vitro Methane Production, In Vitro and In Vivo Nutrient Digestibility, Growth Performance, and Meat Quality in Lithuanian Blackface Lambs

**DOI:** 10.3390/ani16091362

**Published:** 2026-04-29

**Authors:** Tomas Lileikis, Violeta Razmaitė, Virginijus Uchockis, Saulius Bliznikas

**Affiliations:** 1 Department of Animal Nutrition and Feedstuffs, Animal Science Institute, Lithuanian University of Health Sciences, R. Žebenkos 12, 82317 Baisogala, Lithuania; 2 Department of Animal Breeding and Reproduction, Animal Science Institute, Lithuanian University of Health Sciences, R. Žebenkos 12, 82317 Baisogala, Lithuania; 3 Analytical Laboratory, Animal Science Institute, Lithuanian University of Health Sciences, R. Žebenkos 12, 82317 Baisogala, Lithuania

**Keywords:** essential oil, linalool, eugenol, geranyl acetate, geraniol, methane emission, nutrient digestibility, growth performance, meat quality, sheep

## Abstract

Essential oil-based feed additives have been proposed as a practical strategy to mitigate enteric methane emissions in ruminants, but their effects are not always consistent. The objective of this study was to evaluate the effects of dietary supplementation with an essential oil blend on in vitro methane production, rumen fermentation, nutrient digestibility, growth performance, carcass traits, and meat quality in Lithuanian Blackface lambs. We hypothesized that supplementation would induce measurable changes in in vitro methane production and selected rumen fermentation variables, while growth performance and technological meat quality would remain comparable between treatments. Sixty Lithuanian Blackface lambs were allocated to control (C) and treatment (T) groups (30 lambs per group). The C group received a basal diet, and the T group received the same diet supplemented with an essential oil blend, Agolin Ruminant, at a dose rate of 0.1 g/animal/day, consisting of linalool, eugenol, geranyl acetate, and geraniol. An in vitro rumen fermentation assay was performed using rumen fluid pooled within both dietary groups from multiple lambs and incubated as a single batch with four replicate fermentation flasks per treatment (*n* = 4 fermenters per group) to quantify methane production and in vitro nutrient digestibility. In vivo apparent nutrient digestibility was evaluated in a dedicated sub-trial (*n* = 6 animals per group). Growth performance in the main trial was analyzed using the pen as the experimental unit (*n* = 3 pens per group), and slaughter-based measurements—including slaughter and carcass traits, rumen volatile fatty acids and protozoal counts, and *Longissimus dorsi* meat quality and intramuscular fatty acids—were determined in 10 lambs per treatment (*n* = 10 animals per group). In vitro methane production did not differ between groups (*p* = 0.366); in vitro crude fiber digestibility showed a tendency to increase with supplementation (*p* = 0.066). Fermentation end-products were largely unchanged, although propionate tended to be higher (*p* = 0.063), and the acetate:propionate ratio was lower (*p* = 0.043) in the supplemented group; protozoal counts were not different between groups. In vivo apparent nutrient digestibility was comparable between treatments. Growth performance was lower in the supplemented group, resulting in an overall mean ADG 19.0% lower. Slaughter and carcass traits were comparable between treatments. Meat proximate composition, cholesterol concentration, pH, color, water losses, and instrumental texture/shear parameters were not affected by supplementation. Intramuscular lauric (C12:0), myristic (C14:0), and pentadecanoic (C15:0) fatty acids were lower (*p* < 0.05), while C14:1 n-7 tended to decrease (*p* = 0.050); however, total saturated, monounsaturated, and polyunsaturated fatty acids and nutritional ratios were unchanged. Overall, under the study conditions and dose used, the essential oil blend did not significantly reduce in vitro methane production and elicited only limited rumen fermentation responses; ADG was 19.0% lower in the supplemented group, whereas carcass traits and technological meat quality were unaffected, and only specific intramuscular fatty acids were altered.

## Simple Summary

Methane released by sheep during digestion contributes to greenhouse gas emissions, so farmers and researchers are interested in practical ways to reduce it without harming animal performance or product quality. One possible approach is the use of natural feed additives based on essential oils, but their effects are not always consistent. In this study, we evaluated an essential oil blend in Lithuanian Blackface lambs to determine whether it could influence methane production during digestion and affect feed digestibility, growth, and meat quality. The essential oil blend did not produce a reliable reduction in methane in this trial, and in vivo feed digestibility, carcass traits, and most meat quality characteristics were similar between supplemented and control groups. However, lamb growth performance was lower in the supplemented group, resulting in a lower average daily gain over the fattening period. Meat composition, cholesterol, color, moisture retention, and tenderness-related measurements were also comparable. The supplement did, however, slightly alter the levels of a few specific saturated fatty acids in the meat. Overall, the results provide evidence-based information on what can be expected from this type of natural dietary approach under the tested feeding conditions.

## 1. Introduction

Methane (CH_4_) is a potent greenhouse gas (GHG), exhibiting ~84 times greater global-warming potential than carbon dioxide (CO_2_) on a 20-year timescale [1]. Only around 40% of the total CH_4_ emissions come from natural sources (wetlands, termites, and oceans), while 60% are anthropogenic, arising from sources such as ruminant livestock, rice paddies, and landfills [2].

Methanogenesis is a natural part of rumen microbial fermentation, performed by methanogenic archaea that remove excess hydrogen by producing CH_4_. This process not only contributes to atmospheric GHG but also reduces the animal’s energetic efficiency—losses can reach roughly 12% of gross feed energy, depending on diet and production system [3].

Various mitigation strategies have been explored to reduce enteric methane, including manipulation of feeding composition, the use of natural and synthetic compounds, vaccination, and genetic selection, with varying degrees of efficacy across experimental designs. Natural plant-derived compounds—notably essential oils (EOs)—have attracted the attention of researchers as a potentially effective, low-cost, consumer-friendly, and “green” labeled strategy to curb CH_4_ emissions. Essential oils are plant secondary metabolites (PSMs) that primarily protect plants from various stressors, such as pests, infections, and abiotic stress [4]. EOs are complex mixtures consisting mainly of terpenes and terpenoids (oxygenated terpenes) and phenylpropanoids, but other compounds, such as alcohols, aldehydes, ketones, esters, and phenols, can also be present at varying concentrations [5,6,7].

Because EOs can exert antimicrobial properties, they are being studied as potential rumen modifiers and as phytogenic feed additives in livestock [8]. Although the exact mechanisms of action on rumen methanogenesis are not yet precise, it is thought that EOs can affect CH_4_ emissions directly by exhibiting toxic effects on methanogenic archaea or indirectly, by modulating populations of other rumen microorganisms, such as bacteria and protozoa, thus limiting hydrogen (H_2_) availability for methanogenesis [9].

Some controlled trials and recent meta-analyses reported modest improvements in growth performance, feed efficiency, nutrient digestibility, and some meat quality traits following essential oil supplementation [10,11,12,13].

Conversely, other trials and systematic reviews document neutral or dose-dependent adverse effects on feed intake, digestibility, or growth, emphasizing substantial heterogeneity that appears to be linked to essential oil composition, inclusion rate, delivery method, and the basal diet [14,15].

This variability highlights the importance of both dose and composition when evaluating EO-based strategies. In the present study, the supplementation level (0.1 g/animal/day) was in accordance with the manufacturer’s recommendation, and the blend was selected as a commercial formulation with declared major constituents (linalool, eugenol, geraniol, and geranyl acetate). To our knowledge, this formulation has been evaluated primarily in cattle, whereas evidence in sheep remains limited; therefore, testing the blend in Lithuanian lamb production provides species- and setting-specific data relevant to practical use and to the heterogeneous responses reported across studies. The objective of this study was to evaluate the effects of an essential oil blend on in vitro methane production, rumen fermentation characteristics, nutrient digestibility, growth performance, carcass traits, and meat quality in Lithuanian Blackface lambs. We hypothesized that supplementation at 0.1 g/animal/day could lead to measurable changes in in vitro methane production and/or selected rumen fermentation variables, while growth performance and technological meat quality would remain comparable between treatments.

## 2. Materials and Methods

### 2.1. Animals and Housing

The experiment was approved by the Board of the Animal Science Institute of the Lithuanian University of Health Sciences (Protocol No. 25/02/20/01, 20 February 2025).

Sixty healthy Lithuanian Blackface ram lambs were selected for the trial. The study was conducted as a two-group comparative feeding trial, and lambs were assigned to the groups according to body weight and age. The animals were assigned to two groups, control (C) and trial (T), with 30 lambs in each. Following group allocation, lambs were placed in pens of 10 animals each, arranged such that each pen in one group corresponded closely to a pen in the other group in terms of average age and body weight (Table 1).

All lambs were housed in the controlled fattening station of “Genetiniai ištekliai” Ltd. (Upytė, Lithuania) Sheep Farming Division (Pavartyčiai, Lithuania). Animals from both groups were housed in the same naturally ventilated barn under identical environmental conditions—in pens with deep straw bedding, with free access to clean water and salt licks.

### 2.2. Experimental Monitoring

During the trial, lambs were weighed approximately every thirty days to monitor body weight and calculate average daily gain (ADG). After the third scheduled weighing, a subset of lambs was temporarily transferred for the in vivo digestibility sub-trial; following their return to the fattening station, routine weighing was not continued. Therefore, ADG calculations are restricted to the defined measurement period. Lambs nevertheless remained on their respective diets until they reached the target live weight for slaughter (~45 kg). For growth performance analysis, the pen was considered the experimental unit; therefore, individual lamb ADG values were averaged within the pen before statistical analysis.

### 2.3. Feeding, Supplementation, and Feed Analysis

#### 2.3.1. Feeding

Lambs were fed twice daily. The basal ration consisted of perennial grass hay, perennial grass wilted silage (haylage), and a mixture of crushed grains (barley, oats, vetch, and fodder peas) (Table 2). During the first feeding (~09:00), animals received wilted silage, and the grain mixture (250 g); hay and the grain mixture (250 g) were offered during the second feeding (~16:00). The amount of grain mixture provided was 500 g/animal/day. Initially, feed offered, and refusals of wilted silage and hay were recorded for each pen during the trial period to estimate roughage intake. However, accurate quantification of roughage intake was limited due to frequent pulling out of roughages by the lambs and subsequent trampling into the straw bedding. As a result, these data were excluded from the statistical analysis.

#### 2.3.2. Supplementation

After a 14-day adaptation period, a commercial essential oil blend was introduced to the trial group at a dose rate of 0.1 g/animal/day in the grain mixture, in accordance with the manufacturer’s recommended inclusion rate. Lambs were supplemented with a commercial essential oil blend, Agolin Ruminant (Agolin SA, Bière, Switzerland), containing linalool, eugenol, geranyl acetate, and geraniol as the main active compounds. To promote homogeneous distribution, the EO blend was first prepared as a carrier premix by mixing 1 g of EO with 0.5 kg of finely ground flour produced from the same grain mixture. For daily feeding, three premix portions (total: 3 g EO in 1.5 kg carrier) were incorporated into 13.5 kg of the grain mixture to obtain a 15.0 kg total supplemented concentrate batch, corresponding to 0.2 g/kg of essential oil blend in the concentrate. This provided 0.1 g EO/animal/day for 30 lambs receiving 500 g concentrate per day. The supplemented concentrate was prepared fresh each day, thoroughly mixed after stepwise layering of concentrate and premix in a large bin, and offered in two equal meals (250 g each) prior to roughage feeding to minimize storage time.

#### 2.3.3. Feed Analysis

The chemical composition of the feed was analyzed weekly. The evaluated parameters were dry matter (DM), crude protein (CP), ether extract (EE), crude fiber (CF), nitrogen-free extract (NFE), ash, neutral detergent fiber (NDF), and acid detergent fiber (ADF).

### 2.4. In Vivo Nutrient Digestibility Trial

During the main trial, lambs from the control and treatment groups (six from each) were selected for the dietary nutrient digestibility sub-trial, which was performed in the Animal Feedstuff Nutrient Digestibility Research Laboratory of the Department of Animal Nutrition and Feedstuffs. The trial was conducted according to the methodology described by Tomme [16]. The animals were housed individually in metabolic cages and allowed a 5-day adaptation period, followed by a 10-day measurement period. During the study, lambs received the same diet as at the controlled fattening station. However, the amount of wilted silage and hay offered was 800 g and 600 g, respectively, while the concentrate (crushed grain mixture) was provided at 500 g/animal/day. The final nutrient composition of the diet is provided in Table 3.

Feed offered, refusals, and total fecal output were recorded daily throughout the experimental period, and samples were collected for chemical analysis.

### 2.5. Animal Slaughter

During the study period, 10 animals from each group that had reached a weight of ~45 kg were selected for slaughter. The animals were slaughtered at the licensed abattoir of the Sheep Farming Division of “Genetiniai ištekliai” Ltd.

Immediately after slaughter, the rumen digesta samples (*n* = 10) were collected into thermos flasks for subsequent laboratory analyses. The samples were used for in vitro methane emission measurements, estimation of apparent feed digestibility, determination of volatile fatty acid (VFA) concentrations and ratios, and evaluation of protozoa counts.

### 2.6. In Vitro Fermentation Trial

#### 2.6.1. Artificial Saliva

Artificial saliva was prepared 2–3 h before the start of the fermentation, according to McDougall’s method [17].

#### 2.6.2. Experimental Substrate

The feed samples were ground using a sieve with 2 mm diameter holes. The substrate was prepared in the same ratio as fed to the animals. A total of 10.8 g of the ground air-dried feed was placed into nylon in situ bags R1020 (Ankom Technology Corp., Fairport, NY, USA). For the trial group, 0.56 mg of EO was weighed using an analytical scale from Kern ABJ (Kern & Sohn GmbH, Balingen-Frommern, Germany) and added to the bags. Each bag was sealed with a plastic zip tie.

#### 2.6.3. Starting the Fermenter

The in vitro fermentation trial was conducted using the RUSITEC fermentation system (Sanshin Industrial Co. Ltd., Yokohama, Japan) as described by Kajikawa et al. [18]. Each treatment was run using four independent fermenters (*n* = 4 per treatment). The fermenter was considered the experimental unit for methane production and in vitro digestibility, which were determined after 48 h of incubation from the respective gas collection bags and recovered substrate bags. In vitro apparent nutrient digestibility was calculated from the difference between the absolute nutrient mass in the substrate before incubation and in the recovered residue after fermentation, using the measured nutrient concentrations in the initial substrate and in the dried residues.

Before the experiment, a water bath equipped with a TR-1AR heating controller (AS ONE Corp., Osaka, Japan) was filled up to the designated level with distilled water. The heating controller was set to 39 °C. At the start of the experiment, rumen digesta was collected at slaughter into pre-warmed thermos flasks and filtered through four layers of gauze to separate solid and liquid fractions. Because the inoculum was obtained post-slaughter and represents a single sampling point, this is acknowledged as a sampling-related limitation. Throughout filtration and subsequent procedures, CO_2_ was continuously introduced to maintain anaerobic conditions. The inner vessel of each fermenter was inoculated with two nylon bags: one containing the experimental substrate, the other containing 70 g of the solid fraction of the rumen digesta. Each fermenter was filled with 400 mL of the liquid fraction of the rumen digesta and topped up with artificial saliva. The fermenters were sealed with lids and screw-on flanges, installed in the water bath, connected to the rotating motor plate, and linked to artificial saliva input and waste output systems. The FR-D700 inverter (Mitsubishi Electric Manufacturing Co., Ltd., Chiyoda, Tokyo, Japan) was set to 25.00 Hz, corresponding to the recommended motor speed of 4 rpm. A MasterFlex L/S peristaltic pump (Vernon Hills, IL, USA) with Masterflex LS13 tubing was used to deliver the artificial saliva to the fermentation vessels. The pump rotation speed was set to 8 rpm, yielding a flow rate of 0.4 mL/min, thereby maintaining the recommended dilution rate of 3%/h in the fermentation vessels. The waste tube from each fermenter was connected to a waste collection bottle, to which an empty gas collection bag (Tedlar Gas Sampling Bags, Supelco, Bellefonte, PA, USA) was attached. To prevent secondary fermentation in the waste bottle, 7 mL of 35% formaldehyde (calculated for 700 mL of waste) was added in advance.

### 2.7. Meat Quality Assessment

#### 2.7.1. Chemical Analysis

Samples of the *Longissimus dorsi* muscle were collected 24 h after slaughter for chemical composition analysis (DM, CP, EE, Ash), cholesterol content and fatty acid profile analysis, color parameters, water-holding capacity, and texture property evaluation.

#### 2.7.2. Instrumental Evaluation of Texture

Meat texture properties (cohesiveness, gumminess, hardness, springiness, chewiness, work of shear, toughness) were assessed using an Ametek Lloyd TA1 texture analyzer (AMETEK Inc., Berwyn, PA, USA) as described by Razmaitė et al. [19].

#### 2.7.3. Color

Color parameters were measured using a CR-410 Chroma Meter (Konica Minolta, Tokyo, Japan), described by Razmaitė et al. [19]. The evaluated parameters were lightness, L*; redness, a*; yellowness, b*; chroma, C; and hue, h.

#### 2.7.4. Water-Holding Capacity

Water-holding capacity was assessed using the drip loss and cooking loss methods. Drip loss was evaluated using the EZ-DripLoss method [20]. For cooking loss determination, samples were thawed at 4 °C for 24 h, weighed, sealed in thin-walled plastic bags, cooked at 80 °C for 1 h, then cooled to room temperature and reweighed [21].

#### 2.7.5. pH Measurement

The pH of the meat was measured 24 h after slaughter using a PT-380 pH meter (Boeco, Hamburg, Germany).

### 2.8. Methods of Analysis

#### 2.8.1. Chemical Analysis of Feedstuffs and Meat

Total dry matter (DM) content was determined by the gravimetric method, drying samples for 24 h at 60 °C and an average of 3 h (until constant weight) at 105 °C [22].

Crude protein (CP) content was determined by the Kjeldahl method AOAC 984.13, using Tecator (Foss-Tecator AB, Höganäs, Sweden) equipment [22]. To determine the ether extract (EE) content, samples were extracted with petroleum ether (40–60 °C fraction) using a Soxtherm (C. Gerhardt GmbH and Co. KG, Königswinter, Germany) device. After drying, the EE residue was determined gravimetrically. Crude fiber (CF) content was determined using Fiber Cap (Foss-Tecator AB, Höganäs, Sweden) equipment; samples were treated with 0.255 N sulfuric acid and 0.313 N NaOH solutions. Ash content was determined by the gravimetric method, by dry mineralization of samples at 400–500 °C [22]. To determine neutral detergent fiber (NDF) and acid detergent fiber (ADF) content, the ANKOM A200 filter bag technique (FBT) was performed (ANKOM Technology, Macedon, NY, USA), using a neutral solution for NDF and a 1.00 N sulfuric acid solution for ADF. Organic matter (OM) was calculated using the formula OM=DM-Ash, while nitrogen-free extract was calculated using the formula NFE = DM − CP − EE − CF − Ash.

Cholesterol content in meat was determined as described by Polak et al. [23].

Fatty acid composition in meat was determined using methods described by Folch et al. [24] and Christopherson and Glass [25].

#### 2.8.2. Analysis of Gas

The volume of gas produced was determined using a Gas meter (Model DC, Shinagawa Corp., Tokyo, Japan). The bags containing the collected gas were later submitted to a laboratory for CH_4_ content determination using a gas chromatograph (GC-2010 Plus, Shimadzu Corp., Kyoto, Japan) equipped with a thermal conductivity detector (TCD). Methane in the gas mixture was separated on a capillary column CarboPLOT P7 (25 m, ID: 0.53 mm, df: 25 µm) (Agilent Technologies, Middelburg, The Netherlands) by temperature programming from 40 °C to 80 °C. The injector and detector temperatures were held at 80 °C and 100 °C, respectively. The rate of flow of carrier gas (hydrogen) through the column was 36.9 mL/min. The methane peak was identified by comparison with the methane peak in a gas mixture of known composition (Industrial Scientific Oldham, France). The relative proportion of methane was expressed as the relative percentage of the sum of the total gases using LAB Solutions LC/GC (version 5.71) software for Shimadzu gas chromatograph workstations and multiplied by a response factor of 0.028 [26].

### 2.9. Rumen Digesta Parameters

For the pH and VFA analysis, rumen digesta was collected from lambs after slaughter and later strained through 4 layers of gauze. The pH was measured with an Orion 710 pH meter and a glass electrode (Thermo Fisher Scientific, Waltham, MA, USA). For total volatile fatty acid (TVFA) analysis, samples were acidified with 5.00 N H_2_SO_4_ at a ratio of 1:1 and distilled using a Markham apparatus. VFA percentage ratios were measured using a Shimadzu GC-2010 gas chromatograph (Shimadzu Corporation, Kyoto, Japan) fitted with a flame ionization detector (FID) as described by Erwin et al. [27]. VFA were separated on a Stabilwax-DA capillary column (30 m, ID: 0.25 mm, df: 0.2 µm) (Restek, Bellefonte, PA, USA) by temperature programming from 80 °C to 140 °C. The injector and detector temperatures were held at 250 °C. The rate of flow of carrier gas (nitrogen) through the column was 1.18 mL/min. The VFA peaks were identified by comparison with the retention times of a laboratory-prepared fatty acid standard. The relative proportion of each fatty acid was expressed as the relative percentage of the sum of the total fatty acids using LAB Solutions LC/GC (version 5.71) software for Shimadzu gas chromatograph workstations.

Protozoal counts were performed using a Goryaev chamber. Rumen fluid was preserved by mixing the sample at a 1:1 ratio with 4% formalin and gently homogenizing. The fixed suspension was loaded into the chamber and covered with a glass coverslip; correct seating of the coverslip was verified by the appearance of Newton’s rings. The chamber was examined by light microscopy, and protozoa were counted within the chamber grid. Protozoal concentration was calculated using the chamber conversion factor and dilution correction as follows: cells/mL = *N* × 5000, where *N* is the number of protozoa counted in the defined counting area; the factor 5000 accounts for the counted chamber volume and the 1:1 dilution with formalin.

### 2.10. Statistical Analysis

All data were analyzed using the general linear model (GLM) procedure in IBM SPSS Statistics (Version 29.01.0, IBM Corp., Armonk, NY, USA). The GLM univariate model included group (C and T) as a fixed factor. The statistical model was as follows: $Y_{ij}=\;\mu +G_{i}+e_{ij}$, where $Y_{ij}$ is the observed value of the dependent variable, μ is the overall mean, $G_{i}$ is the fixed effect of group (*i* = C, T), and $e_{ij}$ is the residual error. Data distribution within groups was assessed using the Shapiro–Wilk test, and homogeneity of variances was evaluated using Levene’s test. The results of these tests supported the application of parametric analysis. Comparisons were performed using the LSD test. Differences were considered significant at *p* < 0.05, and at 0.05 < *p* < 0.1—as tendencies.

## 3. Results

### 3.1. In Vitro Methane Emissions and Nutrient Digestibility

Methane production did not differ between treatments (*p* = 0.366), although mean values were 4.49 and 6.62 L/1000 g DM/24 h for the essential oil and control groups, respectively. Apparent digestibility of dry matter, crude protein, ether extract, and nitrogen-free extract did not differ between treatments (*p* > 0.05). Crude fiber digestibility tended to be higher in the essential oil-supplemented group (*p* = 0.066). This tendency should be interpreted cautiously and confirmed in further studies (Table 4).

### 3.2. Volatile Fatty Acid Content in Rumen Digesta Samples

Supplementation with the essential oil blend did not significantly affect the VFA profile (% of total VFA), total VFA concentration, or protozoal counts (*p* > 0.05). Propionate tended to be higher in the treated group than in the control (*p* = 0.063), and the acetate:propionate ratio was lower in the treatment group (*p* = 0.043) (Table 5).

### 3.3. In Vivo Nutrient Digestibility

No statistically significant differences were detected between the control and essential oil-supplemented groups for any measured digestibility parameters (*p* > 0.05) (Table 6). Apparent total-tract digestibility of dry matter, organic matter, crude protein, ether extract, crude fiber, and nitrogen-free extract remained comparable between treatments, and the digestibility of the NDF and ADF fractions did not differ significantly. These findings suggest that the essential oil supplementation had no measurable impact on nutrient digestion under the in vivo conditions of this sub-trial; however, given the limited sample size (*n* = 6 per treatment), statistical power was low, and small-to-moderate effects cannot be excluded.

### 3.4. Average Daily Gain

The average daily gain (ADG) of lambs during the experimental period is presented in Table 7. During the first growth period (7–30 June), ADG was significantly lower in the treatment group than in the control group (92 vs. 118 g/day; *p* = 0.007), representing a 22.3% reduction. During the second period (30 June–28 July), ADG was also lower in the supplemented group (109 vs. 123 g/day), although the difference was not significant (*p* = 0.150). In the final period (28 July–25 August), ADG increased in both groups, but remained significantly lower in the supplemented group (158 vs. 203 g/day; *p* = 0.048), corresponding to a 21.9% reduction. Overall, lambs in the supplemented group had significantly lower ADG than control lambs (121 vs. 150 g/day; *p* = 0.008), representing a 19.0% reduction.

### 3.5. Carcass and Meat Quality

#### 3.5.1. Carcass and Slaughter Performance

The carcass and slaughter performance of lambs (Table 8) did not differ between control and treatment (*n* = 10 per group). Live weight at slaughter, age, average daily gain, hot and cold carcass weights, and carcass yield were all similar between groups; treatment-related differences were negligible (≤~2.5%) and statistically non-significant.

#### 3.5.2. Chemical Composition and Cholesterol Content of Muscle

Proximate composition and cholesterol content of the *Longissimus dorsi* muscle are summarized in Table 9. Overall, the meat was lean (intramuscular fat at 1.73% and 1.81% for the control and treatment groups, respectively), with a protein content close to 19% and a dry matter level of ~22–23%, while cholesterol averaged ~49 mg/100 g. Supplementation with the essential oil blend did not alter basic chemical composition or cholesterol: numerical differences between control and treatment were very small (≤~1 percentage point for DM and protein, 0.11 mg/100 g for cholesterol) and were not statistically significant.

#### 3.5.3. Color, pH, and Water-Holding Capacity

Meat pH, instrumental color, and water-holding capacity of the LD muscle are presented in Table 10. No significant differences were observed between groups for pH, instrumental color parameters, drip loss, or cooking loss.

#### 3.5.4. Fatty Acid Composition

Total SFA, MUFA, and PUFA, together with PUFA/SFA, n-3, and n-6 PUFA contents and the n-6/n-3 ratio of intramuscular fat are presented in Table 8. These summary indices were comparable between control and treatment groups; although the n-6/n-3 ratio was higher in the treatment group, none of the differences were statistically significant (Table 11).

The fatty acid composition of intramuscular lipids is presented in Table 12. There were notable reductions in several short- and medium-chain saturated fatty acids—the amounts of lauric (C12:0), myristic (C14:0) and pentadecanoic (C15:0) fatty acids in the intramuscular fat of EO-supplemented lambs decreased by 30.55, 12.88, and 15.63%, respectively (*p* < 0.05). Furthermore, a 15.8% decrease in C14:1 n-7 was also detected, approaching statistical significance (*p* = 0.05). In contrast, the proportions of the major long-chain fatty acids (e.g., palmitic C16:0 and oleic C18:1 n-9) and the summed indices (total trans-FAs, total unidentified FAs, total UFA) were essentially unchanged.

Despite marked reductions in some SFAs, the total SFA amount did not differ between the two groups. Overall, supplementation produced a selective shift in the SFA pattern of intramuscular fat without altering the dominant unsaturated fatty acid profile.

#### 3.5.5. Texture Characteristics

Texture parameters of the *Longissimus dorsi* muscle determined by TPA and Warner–Bratzler shear are presented in Table 13. Cohesiveness, gumminess, hardness, springiness, chewiness, work of shear, and shear toughness showed only minor numerical variation between groups. Springiness tended to be slightly higher, and gumminess and hardness were numerically greater, in the treatment group, whereas work of shear and toughness were slightly variable; none of these differences reached statistical significance.

## 4. Discussion

### 4.1. In Vivo Nutrient Digestibility

In vivo apparent total-tract digestibility of dry matter, organic matter, crude protein, ether extract, crude fiber, nitrogen-free extract, and the NDF and ADF fractions showed no statistically significant differences between the control and essential oil-supplemented lambs. However, the digestibility sub-trial included a limited number of animals (*n* = 6 per treatment), which reduces statistical power; therefore, small-to-moderate treatment effects cannot be excluded, and findings should be interpreted cautiously. Within the heterogeneous literature on essential oils in small ruminants, neutral responses in apparent digestibility have also been reported for blends with different compositions from the EO blend used here. Khateri et al. [28] reported that a thyme–cinnamon–clove essential oil blend did not affect apparent total-tract digestibility of DM, CP, OM, or NDF in sheep fed a 50:50 alfalfa hay: concentrate diet, despite measurable changes in some rumen variables. Likewise, in Hu lambs, supplementation with a cinnamaldehyde–thymol–carvacrol blend had no clear effect on most nutrient digestibility coefficients across doses, with only the highest inclusion level reducing crude protein digestibility while increasing NDF and ADF digestibility [29].

In contrast, several studies in lambs and growing ewes have reported improvements in the digestibility of specific nutrient fractions when other eugenol- or cinnamaldehyde-based blends were supplemented. An oregano essential oil–cobalt blend increased crude protein digestibility in lambs without consistently altering fiber digestibility, and this was accompanied by higher growth performance and carcass weights [30]. A microencapsulated cinnamaldehyde–eugenol–capsicum oleoresin (CEC) blend improved NDF digestibility and average daily gain in growing ewes at higher inclusion levels [31]. Supplementation with *Allium mongolicum* essential oil has also been shown to increase apparent dry matter and crude protein digestibility in sheep [32]. Because results differ across studies, we hypothesize that in vivo nutrient digestibility responses to essential oils in small ruminants depend not only on the active components, dose, and basal diet, but also on the delivery form and dosing pattern (e.g., premix vs. microencapsulated; continuous vs. meal-time exposure), and on potential effects on concentrate intake/palatability and feeding behavior, which can alter the effective intake of the additive and its ruminal exposure. In the present study, the digestibility sub-trial included a limited number of animals (*n* = 6 per group), which reduces statistical power; therefore, small effects of supplementation on digestibility cannot be excluded.

### 4.2. In Vitro Methane Emissions, Nutrient Digestibility, and VFA Profile

Screening studies with individual essential oil active compounds, including linalool, eugenol, and geraniol, have reported substantial reductions in methane at higher inclusion rates in vitro. Still, these decreases were often accompanied by reduced dry matter digestibility or VFA production, indicating a trade-off between methanogenesis inhibition and overall fermentation. For example, Joch et al. [33] reported a 24% reduction in methane production using eugenol in vitro at a dose rate of 1000 μL/L, but total VFA concentration also decreased. In contrast to our research, the authors noted that eugenol decreased the molar proportion of propionate. However, researchers analyzed a trial conducted by colleagues that used a much lower eugenol dose (2.2 mg/L) and reported an increase in the propionate molar fraction, suggesting that differences in the VFA profile might be dose-dependent. In the same in vitro study, Joch et al. [33] also tested linalool and reported a reduction in methane production, accompanied by a decrease in the proportion of propionate and an increase in the proportion of butyrate. In a study where geraniol was used, Joch et al. [34] reported that all three doses used—300, 600, and 900 mg L^−1^—reduced methane production by 10.2, 66.9, and 97.9%, respectively. However, VFA production and DM digestibility were also negatively affected.

In our study, rumen fluid used as inoculum for the in vitro fermentation assay was collected from lambs that had been supplemented with the essential oil blend throughout the fattening period, in accordance with the manufacturer’s recommendation to allow adaptation of the rumen microbiota to the additive. Since direct in vivo measurement of enteric methane was not feasible in this trial, the in vitro experiment used inoculum from adapted animals to ensure that any persistent microbial shifts induced by long-term supplementation were represented in the fermentation system. The underlying expectation was that, if the blend had modified the rumen microbial community in vivo, these changes would also be detectable as alterations in methane production and VFA profiles in vitro. However, because methane was not measured in vivo, the present study cannot directly assess enteric methane mitigation under farm conditions, and methane-related findings should be interpreted as in vitro outcomes only.

Methane production did not differ significantly between the control and essential oil-supplemented groups, indicating that the tested blend at the used dosage (0.1 g/animal/day) did not exert a measurable antimethanogenic effect under the in vitro conditions used. To our knowledge, there are currently no peer-reviewed data on the effects of this specific essential oil blend in sheep, which limits the ability to make direct species-specific comparisons with our findings. Most available information comes from dairy cattle, where the same formulation has reduced methane yield in some long-term studies, suggesting that the antimethanogenic response to this blend is context-dependent rather than consistent across ruminant species and production systems [35]. In the present experiment, several factors may have contributed to the absence of an effect: first, the diet composition and feeding strategy differed from those used in most cattle trials, where the blend is typically supplied in total mixed rations or water [35,36]. Second, the blend was incorporated into the concentrate and delivered twice daily, resulting in rapid consumption and short-term pulses of essential oils rather than a more continuous ruminal supply. This interpretation is supported by dairy cow trials in which the same blend was provided in two grain meals per day, suggesting that the pattern of administration can modify the effective exposure of rumen microbes to the active compounds [37]. Third, the inclusion rate used here may have been insufficient to elicit a clear response under these dietary and dosing conditions. In line with the lack of methane response, the fermentation profile showed only trend-level differences, with propionate tending to be higher in the treatment group (*p* = 0.063), while other fermentation variables remained comparable between groups. Crude fiber digestibility also tended to increase in vitro (*p* = 0.066), but this did not reach statistical significance. Therefore, these trend-level shifts should be interpreted cautiously and do not support a clear or biologically meaningful change in fermentation under the conditions of this study. Protozoal counts remained similar between groups, suggesting no detectable effect of the blend on protozoal populations in this experiment. Taken together, the fermentation profile, fiber digestibility tendency, and protozoal data indicate only minimal changes in the measured rumen-related outcomes, consistent with the absence of a statistically significant difference in methane production in vitro.

### 4.3. ADG and Carcass Parameters

Supplementation with the essential oil blend (0.1 g/animal/day, delivered twice daily with the grain mix) was associated with lower growth performance, whereas carcass characteristics were not influenced. Average daily gain, calculated over the monitored growth interval (7 June to 25 August), was 19.0% lower in the supplemented group. In contrast, live weight at slaughter, hot and cold carcass weights, and dressing percentage did not differ between treatments, indicating that the supplementation regimen did not translate into measurable differences in carcass yield or conformation under the conditions of this study.

Growth responses to essential oils in small ruminants are inconsistent and appear highly dependent on formulation, dose expression (per head vs. per kg DM), basal diet, and delivery strategy. A recent meta-analysis of small ruminants reported an overall small positive effect of essential oils on daily weight gain when studies across species and formulations were pooled, highlighting that beneficial responses can occur in some contexts [12]. However, sheep-focused meta-analytic evidence provides a contrasting perspective: Torres et al. [38] reported an overall reduction in ADG with essential oil supplementation in sheep and identified inclusion level as an important moderator, noting that dietary inclusion levels above ~100 mg/kg DM were associated with reduced ADG. Canbolat et al. [39] observed that garlic essential oil supplementation decreased average daily weight gain in growing lambs during a 63-day feeding trial. A recent review discussing garlic-derived additives also notes that excessive antimicrobial pressure from garlic oil (i.e., dosing above an optimal range) may suppress rumen microbial activity and reduce nutrient utilization, thereby impairing growth performance—emphasizing the dose- and context-dependence of these responses [40]. Although garlic oil is not equivalent to the present blend, such findings demonstrate that growth depression with phytogenic/essential-oil additives is biologically plausible in lambs and is not unprecedented. Overall, results in the literature support the view that essential oils do not consistently improve growth in sheep and, depending on exposure conditions, may be associated with neutral or negative performance outcomes.

Several mechanisms could explain the lower ADG observed in our trial despite largely comparable fermentation endpoints and similar apparent total-tract digestibility between groups. First, a modest reduction in voluntary intake remains a plausible contributor because essential oils can influence palatability and feeding behavior. However, in the dedicated in vivo digestibility sub-trial, where individual feed intake was recorded, DMI did not differ between treatments, suggesting that the lower ADG observed in the main trial was not necessarily driven by a consistent reduction in intake. Nevertheless, because individual intake was not quantified throughout the main finishing period, small differences in intake and/or feeding behavior during parts of the trial cannot be fully excluded without high-resolution intake monitoring. Second, a fixed head-based dose means that the effective dietary concentration (mg/kg DM) varies with individual DMI; thus, even at the same nominal dose (0.1 g/animal/day), some animals may experience exposures closer to ranges where sheep meta-analytic evidence has identified performance depression [38]. Third, although the blend was offered twice daily with the grain mix, ruminal exposure may still have been episodic and aligned with feeding events; if active compounds are rapidly absorbed, metabolized, or diluted in the rumen environment, the net effect on energy retention for growth may differ from what is suggested by endpoint rumen measurements. These considerations support a cautious interpretation that the observed reduction in ADG may reflect intake/exposure dynamics and/or subtle shifts in nutrient partitioning that were not captured by the measured fermentation endpoints or apparent total-tract digestibility.

A methodological limitation relevant to growth interpretation is that live weight in the main trial was recorded only up to the last scheduled growth assessment, while lambs remained on their respective diets until slaughter. Therefore, the reported ADG reflects only the monitored interval and does not permit evaluation of late-phase growth dynamics or possible compensatory growth, which has been described in lambs under some conditions [41]. Future studies should include more frequent weighing through to slaughter.

### 4.4. Proximate Composition, Cholesterol, and Fatty Acid Content of Meat

In the present study, supplementation with the essential oil blend did not affect the basic chemical composition of the *Longissimus dorsi* muscle or cholesterol concentration, as dry matter, crude protein, intramuscular fat, ash, and cholesterol showed only minor numerical variation and no significant differences between treatments. Likewise, the overall fatty acid classes and nutritional ratios were unchanged, with similar proportions of SFA, MUFA, and PUFA, and comparable PUFA/SFA, n-3 PUFA, n-6 PUFA, and n-6/n-3 values in both groups. However, despite stable summary indices, the blend induced a selective shift in individual fatty acids, most clearly reflected by lower proportions of several short- and medium-chain SFA (C12:0, C14:0 and C15:0; *p* < 0.05). This pattern—minimal effects on proximate composition and overall FA ratios, but occasional changes in specific fatty acids—has also been observed in lamb studies using other essential oil formulations, where meat chemical composition was largely unchanged while fatty acid responses were limited to particular fractions [42,43,44,45]. More broadly, data from meta-analysis in small ruminants by previous authors [12] suggests that essential oils generally have little influence on meat proximate composition, whereas effects on lipid composition are inconsistent and context-dependent, varying with active compound profile, dose, and basal diet. Although the underlying mechanisms were not directly assessed in the present study, the selective nature of the observed changes is consistent with the possibility that essential oils may influence specific steps of rumen lipid metabolism (e.g., biohydrogenation), resulting in changes in particular fatty acids without necessarily altering total SFA, MUFA, or PUFA in muscle [42,46,47]. Additional non-mutually exclusive explanations may also be considered. For example, essential oils could influence host lipid metabolism, including de novo lipogenesis, potentially affecting the relative abundance of short- and medium-chain SFA [48,49]. The reduction in C15:0 may also reflect subtle changes in rumen microbial lipid synthesis, given that odd-chain fatty acids are commonly linked to microbial metabolism [50,51]. Finally, because intramuscular fat content was low (<2%), small absolute shifts may appear as relatively large proportional differences [52,53]. These interpretations remain speculative and require confirmation in future studies.

From a nutritional perspective, the selective reductions in C12:0 and C14:0 can be viewed as directionally favorable; however, because overall fatty acid classes (SFA, MUFA, PUFA) and nutritional ratios were unchanged, the practical impact on the nutritional value of lamb meat is likely modest.

### 4.5. pH, Color, Water-Holding Capacity, and Texture of Meat

Across the technological quality traits of meat, the essential oil blend produced no measurable changes in LD muscle pH, color, water-holding capacity, or texture. Ultimate pH was virtually identical between treatments (5.82 vs. 5.85), and the instrumental color indices (L*, a*, b*, C*, h°) were likewise stable. Drip loss was numerically lower in the treated lambs (2.52% vs. 3.46%), but cooking loss did not differ between groups. Texture profile analysis and Warner–Bratzler outcomes also remained comparable, with a tendency toward higher springiness (*p* = 0.076) in the supplemented group. Similar responses to lamb meat physical traits have been reported when essential oils were supplied via different routes (e.g., drenching with sage/clove/laurel), with effects more evident on oxidative stability during storage than on baseline pH/color/WHC [54,55]. The generally small effect is consistent with evidence that dose and delivery pattern can be more decisive than “EO vs. no EO” per se—e.g., rosemary essential oil altered some meat attributes in a manner that depended on administration form (oral vs. mixed) [56]. In contrast, more pronounced improvements in some meat quality endpoints have been observed under challenge conditions (e.g., heat stress), such as dose-dependent effects of clove essential oil on WHC and shear force [48], reinforcing the context-dependence noted in the small ruminant EO literature [57].

## 5. Study Limitations

Several limitations should be considered when interpreting the present findings. Growth performance was analyzed with pen as the experimental unit, and treatment comparisons were therefore based on three pens per treatment. In vitro fermentation responses were evaluated using four replicate fermenters per treatment, while in vivo apparent nutrient digestibility was assessed with six animals per treatment. Accordingly, the ability to detect small treatment effects may have been limited in some parts of the study. In addition, methane-related responses were evaluated in vitro rather than by direct in vivo methane measurements, which limits direct extrapolation to in vivo emissions under production conditions. Furthermore, a single supplementation dose was evaluated, and thus, potential dose-dependent responses could not be determined. Finally, because live weight was not monitored repeatedly until slaughter, late-phase growth dynamics and possible compensatory growth could not be fully assessed.

## 6. Conclusions

Under the conditions of this study, supplementation with a commercial essential oil blend “Agolin Ruminant” at a dose of 0.1 g/animal/day during the fattening of Lithuanian Blackface lambs did not influence in vitro methane production and had limited effects on rumen fermentation, nutrient digestibility, slaughter characteristics, and technological meat quality traits, while average daily gain was 19.0% lower in the supplemented group and only selective differences were observed in individual intramuscular fatty acids, with no changes in the main fatty acid classes.

Overall, with the tested dose and delivery method, the essential oil blend exhibited limited biological effects and did not mitigate methane or improve performance under the conditions of this study. Further research is needed to evaluate a wider range of inclusion levels and alternative delivery approaches (e.g., encapsulated formulations or strategies that provide more continuous ruminal exposure) to determine whether different dosing and administration patterns can produce more consistent effects on rumen fermentation, methane-related outcomes, and animal performance, without compromising growth or efficiency.

## Figures and Tables

**Table 1:** The average weight and age of animals in each pen at the start of the trial.

Pen	Average Weight, kg	Average Age, d
C1	24.46	135.8
T1	25.67	134.5
C2	25.19	111.8
T2	26.30	110.5
C3	24.39	90.9
T3	24.88	92.9

Values are pen means; *n* = 10 lambs per pen (total *n* = 60). C, control (basal diet); T, treatment group.

**Table 2:** Average chemical composition of feed components used during the main trial.

	Feed Component		
Variable	Wilted Silage	Hay	Concentrate Mix
DM	67.69	83.67	85.96
CP	9.86	6.94	15.02
EE	1.87	1.21	1.42
CF	38.33	37.63	4.78
NFE	43.33	48.93	76.68
Ash	6.62	5.30	2.11
NDF	64.62	68.27	25.16
ADF	43.96	44.16	8.45

DM: dry matter; CP: crude protein; EE: ether extract; CF: crude fiber; NFE: nitrogen-free extract; NDF: neutral detergent fiber; ADF: acid detergent fiber. DM is expressed as % of as-fed; all other values are expressed as % of DM.

**Table 3:** Chemical composition of feed components and calculated composition of the offered ration during the in vivo nutrient digestibility sub-trial.

Variable	Feed Component			Offered Ration, g/kg DM	Total Offered, g/Day
	Wilted Silage	Hay	Concentrate Mix		
DM	66.75	74.97	85.78	-	1412.7
OM	94.51	94.76	97.80	955.9	1350.4
CP	10.26	6.81	15.31	106.9	151.1
EE	2.17	1.52	1.72	18.3	25.8
CF	38.41	41.43	4.69	291.3	411.6
NFE	43.68	45.00	76.08	539.4	762.0
Ash	5.49	5.24	2.20	44.1	62.3
NDF	71.00	77.35	29.44	604.0	853.3
ADF	47.32	49.00	9.89	364.9	515.5

DM, dry matter; OM, organic matter; CP, crude protein; EE, ether extract; CF, crude fiber; NFE, nitrogen-free extract; NDF, neutral detergent fiber; ADF, acid detergent fiber. DM is expressed as % of as-fed (fresh matter); all other feed component values are expressed as % of DM. “Total offered” values are expressed as g/day and were calculated from the fixed daily offers of wilted silage (800 g as-fed), hay (600 g as-fed), and concentrate mix (500 g as-fed) after conversion to DM.

**Table 4:** In vitro methane production and nutrient digestibility of control and essential oil-supplemented diets.

Variable	C *n* = 4	T *n* = 4	SED	*p*-Value
CH_4_ L/1000 g DM	6.62	4.49	2.140	0.366
Digestibility:				
DM	59.31	60.12	2.233	0.731
CP	34.38	32.66	2.817	0.568
EE	36.94	35.64	2.264	0.591
CF	56.27	60.45	1.784	0.066
NFE	69.66	68.34	4.410	0.777

C, control; T, essential oil-supplemented diet; CH_4_, methane; DM, dry matter; CP, crude protein; EE, ether extract; CF, crude fiber; NFE, nitrogen-free extract; SED, standard error of the difference between means. Values are means; *n* = 4 replicate fermentation flasks (fermenters) per treatment. The fermentation flask was considered the experimental unit. Differences between treatments were tested using univariate GLM with treatment as the fixed effect. Statistical significance was declared at *p* < 0.05 and trends at 0.05 < *p* < 0.10.

**Table 5:** pH, volatile fatty acid composition, total VFA concentration, and protozoal count in rumen digesta of lambs.

Variable	C *n* = 10	T *n* = 10	SED	*p*-Value
pH	6.65	6.70	0.163	0.791
Acetate	70.00	66.73	1.994	0.113
Propionate	15.64	18.66	1.523	0.063 ^†^
A:P ratio	4.56	3.76	0.367	0.043 *
Isobutyrate	1.09	0.96	0.217	0.541
Butyrate	10.34	10.41	0.908	0.933
Isovalerate	1.585	1.413	0.347	0.627
Valerate	0.88	1.52	0.389	0.125
Hexanoate	0.404	0.312	0.060	0.141
Total VFA mmol/100 mL	9.82	8.72	0.900	0.237
Protozoa (cells/mL × 10^5^)	7.88	7.73	1.17	0.905

C, control; T, treatment; VFA, volatile fatty acids; SED, standard error of the difference between means. Values are means; *n* = 10 lambs per treatment. Individual VFAs are expressed as % of total VFA; total VFA is expressed as mmol/100 mL. Protozoa are expressed as cells/mL × 10^5^. Differences between treatments were tested using univariate GLM with treatment as the fixed effect (animal as the experimental unit). Significance: ^†^ 0.05 ≤ *p* < 0.10; * *p* < 0.05.

**Table 6:** DMI and nutrient digestibility of control and essential oil-supplemented lamb diets in the in vivo sub-trial.

Variable	C *n* = 6	T *n* = 6	SED	*p*-Value
DMI, kg	1.112	1.120	0.044	0.872
DM	64.48	64.18	1.927	0.880
OM	66.04	66.01	1.905	0.987
CP	52.17	52.03	1.679	0.933
EE	57.14	60.31	4.367	0.484
CF	55.93	56.90	3.623	0.794
NDF	57.38	59.65	3.566	0.538
ADF	55.69	54.75	3.139	0.770
NFE	73.85	73.38	1.452	0.753

C, control; T, treatment; DMI, dry matter intake; DM, dry matter; OM, organic matter; CP, crude protein; EE, ether extract; CF, crude fiber; NDF, neutral detergent fiber; ADF, acid detergent fiber; NFE, nitrogen-free extract; SED, standard error of the difference between means. Values are means; *n* = 6 lambs per treatment. DMI is expressed as kg DM/day. Digestibility coefficients are expressed as % (apparent total-tract digestibility). Differences between treatments were tested using univariate GLM with treatment as the fixed effect (animal as the experimental unit). Statistical significance was declared at *p* < 0.05; values of 0.05 < *p* < 0.10 were considered as tendencies.

**Table 7:** Average daily gain (g/day) of lambs during different growth periods, expressed as pen means.

Period	C *n* = 3	T *n* = 3	SED	*p*-Value
7–30 June	118	92	5.13	0.007 **
30 June–28 July	123	109	8.16	0.150
28 July–25 August	203	158	15.69	0.048 *
Overall	150	121	5.75	0.008 **

C, control; T, treatment; SED, standard error of the difference between means. Values are means; pen was the experimental unit, with *n* = 3 pens per treatment. Differences between treatments were tested using univariate GLM with treatment as the fixed effect. Significance: * *p* < 0.05; ** *p* < 0.01.

**Table 8:** Carcass and slaughter performance of lambs (control vs. treatment).

Variable	C *n* = 10	T *n* = 10	SED	*p*-Value
Live weight, kg	44.7	45.0	0.591	0.635
Age, days	269.4	276.5	8.803	0.430
Daily gain, g	117.4	117.2	6.707	0.976
Hot carcass weight, kg	18.0	18.1	0.330	0.732
Cold carcass weight, kg	17.3	17.7	0.332	0.279
Carcass yield, %	40.2	40.2	0.726	0.967

C, control; T, treatment; SED, standard error of the difference between means. Values are means; *n* = 10 lambs per group. Differences between groups were tested using univariate GLM with group as the fixed effect (animal as the experimental unit). Statistical significance was declared at *p* < 0.05; values of 0.05 < *p* < 0.10 were considered as tendencies.

**Table 9:** Proximate composition and cholesterol content of the LD muscle of lambs.

Variable	C *n* = 10	T *n* = 10	SED	*p*-Value
DM, %	22.26	23.19	0.876	0.300
CP, %	18.80	19.65	0.747	0.273
EE, %	1.73	1.81	0.316	0.791
Ash, %	1.02	1.05	0.048	0.554
Cholesterol, mg/100 g	48.94	49.05	1.151	0.919

C, control; T, treatment; LD, *Longissimus dorsi*; DM, dry matter; CP, crude protein; EE, ether extract; SED, standard error of the difference between means. Values are means; *n* = 10 lambs per group. Differences between groups were tested using univariate GLM with group as the fixed effect (animal as the experimental unit). Statistical significance was declared at *p* < 0.05; values of 0.05 ≤ *p* < 0.10 were considered tendencies.

**Table 10:** pH, color parameters, and water-holding capacity of LD muscle of slaughtered lambs (control vs. treatment).

Variable	C *n* = 10	T *n* = 10	SED	*p*-Value
pH	5.82	5.85	0.030	0.244
L*	49.1	49.28	1.349	0.946
a*	21.30	20.83	0.545	0.400
b*	2.11	2.30	0.445	0.684
C*	21.42	21.02	0.569	0.487
h°	5.58	6.20	1.102	0.584
Drip loss, %	3.46	2.52	0.663	0.175
Cooking loss, %	35.97	36.68	1.230	0.567

C, control; T, treatment; LD, *Longissimus dorsi*; SED, standard error of the difference between means. L*, lightness; a*, redness; b*, yellowness; C*, chroma; h°, hue angle. Values are means; *n* = 10 lambs per group. Differences between groups were tested using univariate GLM with group as the fixed effect (animal as the experimental unit). Statistical significance was declared at *p* < 0.05; values of 0.05 < *p* < 0.10 were considered tendencies.

**Table 11:** Total saturated, monounsaturated, polyunsaturated fatty acids (in %) and fatty acid ratios in the intramuscular fat of the LD muscle of lambs.

Variable	C *n* = 10	T *n* = 10	SED	*p*-Value
SFA	42.266	42.139	0.634	0.843
MUFA	42.360	42.699	0.965	0.729
PUFA	11.798	11.609	0.879	0.832
PUFA/SFA	0.281	0.276	0.024	0.861
n-3 PUFA	2.014	1.866	0.151	0.339
n-6 PUFA	9.784	9.743	0.750	0.956
n-6/n-3 PUFA	4.893	5.249	0.221	0.125

C, control; T, treatment; LD, *Longissimus dorsi*; SFA, saturated fatty acids; MUFA, monounsaturated fatty acids; PUFA, polyunsaturated fatty acids; SED, standard error of the difference between means. Fatty acids are expressed as % of total identified fatty acids. Values are means; *n* = 10 lambs per group. Differences between groups were tested using univariate GLM with group as the fixed effect (animal as the experimental unit). Statistical significance was declared at *p* < 0.05; values of 0.05 < *p* < 0.10 were considered tendencies.

**Table 12:** Fatty acid composition (% of total identified FA) in intramuscular fat of lambs.

Fatty Acid	C *n* = 10	T *n* = 10	SED	*p*-Value
C10:0	0.126	0.113	0.010	0.226
C12:0	0.108	0.075	0.010	0.004 **
C14:0	1.731	1.508	0.071	0.006 **
C15:0	0.256	0.216	0.015	0.014 *
C15:1	0.103	0.101	0.021	0.912
C16:0	21.140	21.044	0.362	0.793
C17:0	0.986	0.944	0.024	0.098
C18:0	17.392	17.722	0.445	0.468
C20:0	0.112	0.106	0.007	0.429
C21:0	0.196	0.188	0.014	0.564
C22:0	0.219	0.223	0.019	0.848
C14:1 n-7	0.139	0.117	0.010	0.050 ^†^
C16:1 n7t	0.532	0.524	0.032	0.809
C16:1 n-9	0.707	0.657	0.062	0.435
C16:1 n-7	1.321	1.211	0.071	0.137
C17:1 n-9	0.549	0.529	0.018	0.267
C18:1 n-9t	1.799	1.463	0.220	0.143
C18:1 n-9	35.981	36.858	0.947	0.367
C18:1 n-7	1.151	1.161	0.065	0.881
C20:1 n-9	0.078	0.079	0.003	0.842
C18:2 n-6t	0.147	0.161	0.040	0.735
C18:2 n-6c, t	0.239	0.242	0.038	0.936
C18:2 n-6t, c	0.251	0.297	0.024	0.065
C18:2 n-6	6.899	6.731	0.501	0.741
C18:3 n-3	1.227	1.129	0.084	0.255
C20:3 n-6	0.160	0.157	0.019	0.878
C20:4 n-6	2.027	2.082	0.274	0.843
C20:5 n-3	0.277	0.266	0.039	0.784
C22:4 n-6	0.061	0.073	0.015	0.448
C22:5 n-3	0.404	0.376	0.049	0.568
C22:6 n-3	0.107	0.097	0.017	0.549
Total TFA	2.968	2.687	0.262	0.298
Total UFA	3.575	3.553	0.421	0.957

C, control; T, treatment; FA, fatty acid; SED, standard error of the difference between means. Fatty acids are expressed as % of total identified fatty acids. c, cis; t, trans. Values are means; *n* = 10 lambs per group. Differences between groups were tested using univariate GLM with group as the fixed effect (animal as the experimental unit). Statistical significance was declared at *p* < 0.05; values of 0.05 ≤ *p* < 0.10 were considered tendencies; ^†^ 0.05 ≤ *p* < 0.10; * *p* < 0.05; ** *p* < 0.01.

**Table 13:** Texture parameters of the LD muscle determined by texture profile analysis (TPA) and Warner–Bratzler shear test.

Variable	C *n* = 10	T *n* = 10	SED	*p*-Value
Cohesiveness	2.06	2.10	0.130	0.710
Gumminess, N	27.69	38.37	10.829	0.337
Hardness, N	27.17	29.91	3.605	0.458
Springiness	0.83	0.85	0.006	0.076
Chewiness, N	35.57	40.48	11.778	0.682
Work of shear, N·m	3.07	2.97	0.216	0.665
Toughness, N	224.48	259.94	88.149	0.692

C, control; T, treatment; SED, standard error of the difference between group means. Values are means; *n* = 10 lambs per group. Differences between groups were tested using univariate GLM with group as the fixed effect (animal as the experimental unit). Statistical significance was declared at *p* < 0.05; values of 0.05 ≤ *p* < 0.10 were considered tendencies.

## Data Availability

The original contributions presented in this study are included in the article. Further inquiries can be directed to the corresponding author.

## Abbreviations

The following abbreviations are used in this manuscript:

CH_4_	Methane
GHG	Greenhouse gas
CO_2_	Carbon dioxide
EO	Essential oil
H_2_	Hydrogen
C	Control
T	Trial
ADG	Average daily gain
DM	Dry matter
CP	Crude protein
EE	Ether extract
CF	Crude fiber
NFE	Nitrogen-free extract
NDF	Neutral detergent fiber
ADF	Acid detergent fiber
VFA	Volatile fatty acid
FBT	Filter bag technique
TVFA	Total volatile fatty acids
GLM	General linear model
IMF	Intramuscular fat
LD	Longissimus dorsi
WHC	Water-holding capacity
SFA	Saturated fatty acids
MUFA	Monounsaturated fatty acids
PUFA	Polyunsaturated fatty acids
TFA	Trans fatty acids
UFA	Unidentified fatty acids
SED	Standard error of the difference between means
TPA	Texture profile analysis
OM	Organic matter
CEC	Cinnamaldehyde–eugenol–capsicum–oleoresin
